# Popliteal Venous Aneurysms: A Systematic Review of Treatment Strategies and Outcomes

**DOI:** 10.3390/jcm14103296

**Published:** 2025-05-09

**Authors:** Ottavia Borghese, Domenico Pascucci, Nicolò Peluso, Francesco Sposato, Antonino Marzullo, Tommaso Donati, Laura Rascio, Yamume Tshomba

**Affiliations:** 1 Unit of Vascular Surgery, Fondazione Policlinico Universitario A. Gemelli IRCCS, 00168 Rome, Italy; ottavia.borghese@guest.policlinicogemelli.it (O.B.); nicopeluso@yahoo.com (N.P.); dott.francescosposato@gmail.com (F.S.); ninimarzullo70@gmail.com (A.M.); tommaso.donati@gmail.com (T.D.); yamume.tshomba@policlinicogemelli.it (Y.T.); 2 Section of Hygiene, Department of Life Science and Public Health, Università Cattolica del Sacro Cuore, 00168 Rome, Italy; domenico.pascucci@policlinicogemelli.it; 3 Health Management, Fondazione Policlinico Universitario A. Gemelli IRCCS, 00168 Rome, Italy; 4 Unit of Vascular Surgery, Department of Cardiovascular Sciences, Università Cattolica del Sacro Cuore, 00168 Rome, Italy

**Keywords:** venous aneurysm, pulmonary embolism, deep venous thrombosis (DVT), popliteal vein aneurysm, lower limb vein aneurysm

## Abstract

**Background:** Popliteal venous aneurysms (PVA) are an uncommon but potentially severe condition due to their association with increased risk of recurrent pulmonary embolisms. Because of their rarity, their aetiology, natural history, and optimal treatment strategies have been poorly defined. The aim of this paper is to report a comprehensive systematic review on the treatment strategies and outcomes in PVA, summarizing current evidence. **Methods:** A systematic literature search was conducted in PubMed, Scopus, and Web of Science, covering studies published from database inception through February 2025 (protocol registered on PROSPERO CRD420251008927). The primary endpoint was the analysis of outcomes and complications associated with surgical and conservative management. **Results:** Nine studies, including 173 adult patients with popliteal venous aneurysms, were included. The mean age was 56 years (range 18–86 years, mean aneurysm diameter 25.4 mm). Most of the patients were female (73, 42.2%). Overall, 85 (49.1%) aneurysms were saccular and 74 (42.8%) fusiform, although morphology was not consistently reported across all studies. Intraluminal thrombus was reported in 26 cases (15.0%), and pulmonary embolism upon presentation in 21 (12.1%). Surgical treatment was performed in 119 patients (68.8%), while 54 (31.2%) were managed conservatively. Fifteen patients (13.0%) experienced postoperative complications, including wound infections (4, 3.5%), hematomas (7, 6.0%), and nerve injury (4, 3.5%), but no cases of postoperative pulmonary embolisms were observed. Following surgery, anticoagulation was indicated in most cases for 3–6 months or a long life. During follow-up (mean 35 months, range 1–262), thrombosis of the surgical reconstruction was observed in 1 patient (0.8%). Death occurred in 3 cases (5.5%), all in the non-surgical group: 2 (3.7%) due to malignancy and 1 (1.9%) from myocardial infarction. **Conclusions:** PVA is a rarely described condition potentially associated with the risk of PE. In their management, surgical strategies in association with oral anticoagulation represent the most commonly described approach, allowing for satisfactory results and a low rate of complications.

## 1. Introduction

Venous aneurysms are uncommon vascular abnormalities defined as a focal dilation two to three times the size of the normal vein diameter [1,2]. Peripheral venous aneurysms (PVAs) are more frequently described in popliteal vessels with a reported prevalence of 0.06% among patients with underlying venous disorders [3]. They may be asymptomatic and incidentally discovered, but most of the cases are diagnosed following thromboembolic complications [1]. The natural history and aetiology of PVAs remain poorly understood due to their rarity.

The pathogenesis of popliteal venous aneurysms remains unclear. Congenital weakness of the venous wall, trauma, inflammation, and local hemodynamic factors have been suggested as possible causes [1].

Proposed etiological factors include trauma, inflammation, and congenital weakness of the venous wall [1]. Importantly, PVAs are recognized as a rare but significant source of pulmonary embolism (PE), which can be the first clinical manifestation. According to current literature, conservative management (clinical observation alone or anticoagulation) may be performed in selected cases, but due to the high risk of pulmonary embolism (PE), a surgical treatment is normally performed [1,2,3,4]. The aim of this paper is to report a comprehensive systematic review on the treatment strategies of this rare condition and associated outcomes, summarizing current evidence and providing a reference for future clinical practice.

## 2. Materials and Methods

### 2.1. Study Design and Literature Search

**Figure 1:**
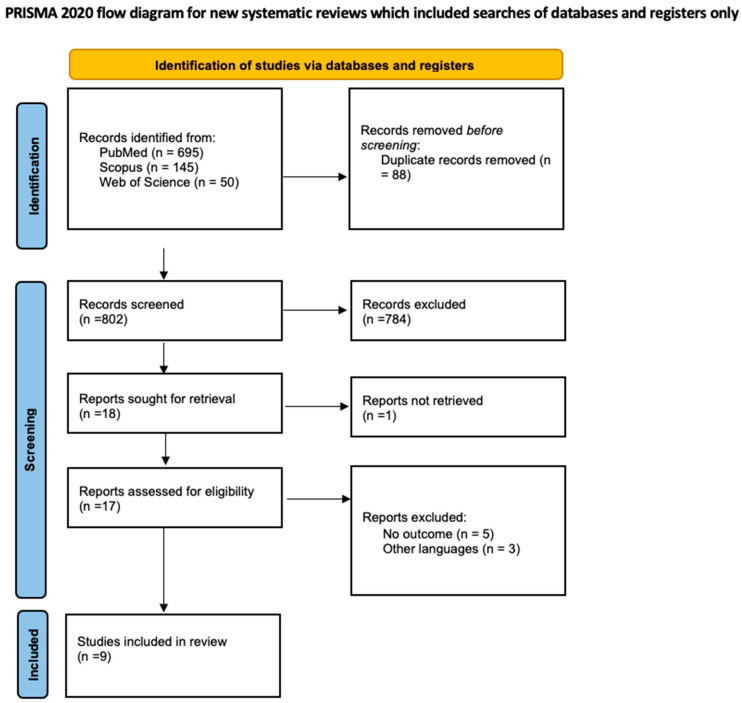
PRISMA 2020 flow diagram adapted from Page et al. (2021) [Page MJ, McKenzie JE, Bossuyt PM, Boutron I, Hoffmann TC, Mulrow CD, et al. The PRISMA 2020 statement: an updated guideline for reporting systematic reviews. BMJ 2021;372:n71. doi:10.1136/bmj.n71]. Available online: http://www.prisma-statement.org/ (accessed on 4 March 2025).

A systematic literature review was conducted using Medline (PubMed.gov, U.S. National Library of Medicine, National Institutes of Health), Scopus, and Web of Science, with the aim of identifying reported cases of PVAs published from database inception through February 2025. Data about the different treatment approaches and outcomes were collected. The research question was structured using the Population, Intervention, Comparison, and Outcomes (PICO) model, focusing on surgical and medical treatment and the reported outcomes. (Table 1) The study included adult patients (≥18 years) of both sexes diagnosed with popliteal venous aneurysms, with reported treatment strategies (surgical or conservative) and associated clinical outcomes. Studies not written in English, case reports with fewer than three patients, review articles, and conference abstracts were excluded. Patients who underwent surgical interventions (aneurysmorrhaphy or bypass with a vein graft) or conservative management were included, and clinical success (resolution of symptoms), recurrence rates, thrombosis of the vascular reconstruction, and complications (pulmonary embolism and local complications) observed during follow-up were analyzed. To ensure methodological rigor and transparency, we followed the Preferred Reporting Items for Systematic Reviews and Meta-Analyses (PRISMA) guidelines, using both the checklist and flow diagram [5,6,7] (Figure 1 and Table S1). The search strategy was built by combining keywords such as “popliteal venous aneurysms”, “venous aneurysms”, and “lower limb venous aneurysms”, using Boolean operators “AND” and “OR”. Additional studies were identified through manual reference screening of the articles included in the review. The protocol for this systematic review was registered on PROSPERO (CRD420251008927) prior to data extraction and analysis.

### 2.2. Study Selection

The screening process for titles and abstracts was carried out independently by two reviewers (LR and NP) to identify potentially relevant studies. Any discrepancies in the selection process were resolved through discussion with a third reviewer (OB). Only articles written in English and containing specific data on patients’ demographics, aneurysm characteristics—including location, size, treatment indications, and symptoms, with particular attention to pulmonary embolism—were included.

### 2.3. Quality Assessment

Two independent investigators (LR and NP) assessed the methodological quality of the included studies using the National Institutes of Health (NIH) Quality Assessment Tools, selected according to study design. For the first eight studies, the NIH Quality Assessment Tool for Observational Cohort and Cross-Sectional Studies—comprising 14 items—was used. For the remaining study, which was classified as a case series, the NIH Quality Assessment Tool for Case Series (9 items) was applied. Each tool evaluates the internal validity of a study through a series of predefined criteria. For each item, reviewers could assign one of three responses: “yes”, “no”, or “cannot determine/not reported/not applicable”. A potential risk of bias was considered present when a criterion was marked as either “no” or “cannot determine/not reported/not applicable” [8]. Based on the proportion of “yes” responses, studies were classified into three levels of methodological quality: “good” (≥75% yes responses), “fair” (50–74%), and “poor” (<50%) [9].

### 2.4. Statistical Analysis

Statistical analysis was performed using SPSS software (IBM Corp., Armonk, NY, USA). Categorical variables are presented as absolute numbers and percentages, while continuous variables are reported as mean and range. Due to the limited number of cases and the high heterogeneity in reporting across studies, no pooled statistical analysis or comparative testing was conducted. A narrative synthesis of the data was reported.

## 3. Results

### 3.1. Quality Assessment

A total of 9 studies met the inclusion criteria and were included in the review. Methodological quality was assessed using two different NIH quality assessment tools, selected based on study design. For the first eight studies, which were review-type or observational studies, we applied the NIH tool for systematic reviews and observational cohorts. For the final study, classified as a case series, the NIH tool specifically developed for case series (comprising 9 items) was used. Overall, 7 studies were rated as fair quality, 1 as poor, and 1 as good. The study rated as good met the majority of the criteria on the case series assessment tool. Across the included studies, the most consistently fulfilled criteria were the clarity of study objectives and adequate patient selection. However, methodological limitations were frequently observed, including lack of randomization and control groups, incomplete or inconsistently reported follow-up data, underreporting of complications, and variability in preoperative data collection. These factors may introduce bias, particularly in the evaluation of long-term outcomes and in the comparison of management strategies. Given these limitations, the results of this review should be interpreted with caution (Table 2).

### 3.2. Study Selection

The literature search yielded 890 records. After removing 88 duplicates, 802 titles and abstracts were screened. Following the exclusion of 784 records, 18 full-text articles were assessed. Of these, 9 studies met the eligibility criteria and were included in the final review.

### 3.3. Demographics

A total of 173 patients with popliteal venous aneurysms were included. The mean age was 56 years (range 18–86 years). Most of the patients were female (73, 42.2%). Comorbidities included hypertension in 30 patients (17.3%), diabetes mellitus in 3 patients (1.7%), and other unspecified cardiac diseases in 6 patients (3.5%). Chronic venous insufficiency was reported in 78 patients (45%), and venous ulcers in 4 patients (2.3%). Previous deep vein thrombosis was reported in 11 patients (6.4%). A history of previous leg trauma or knee injury was documented in 8 patients (4.6%), and prior varicose vein surgery in 7 patients (4.0%). One patient (0.6%) had a history of prior meniscal surgery.

At the diagnosis, 20 patients (11.6%) were asymptomatic, while in symptomatic patients, leg edema or swelling was described in 27 (15.6%) cases, pain in 31 (17.9%), limb discomfort in 4 (2.3%), and a palpable popliteal mass in 1 patient (0.6%). Pulmonary embolism was reported in 21 patients (12.1%), of whom 2 (1.2%) had cardiac arrest and 1 (0.6%) underwent surgical embolectomy.

### 3.4. Aneurysms Characteristics

Regarding aneurysm morphology, 85 (49.1%) were saccular and 74 (42.8%) were fusiform, although morphology was not consistently reported across all studies. Intraluminal thrombus was present in 26 cases (15.0%). The mean aneurysm diameter was 25.4 mm.

**Figure 2:**
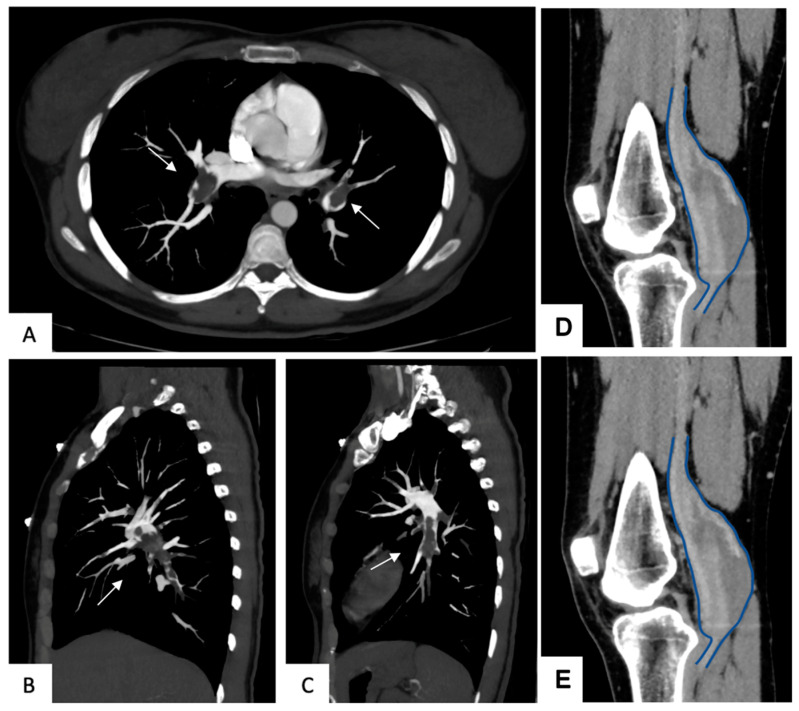
Computed Tomography Angiography (CTA) showing a massive pulmonary embolism (arrow) in the transverse (**A**) and sagittal plane in the right (**B**) and left pulmonary (**C**) in a patient affected with a huge popliteal vein aneurysm treated at our Institution with intraluminal thrombus at the preoperative CTA in axial (**D**) and sagittal (**E**) planes.

All patients were evaluated by duplex ultrasound. In four studies, preoperative venography was also performed (Table 3; Figure 2).

### 3.5. Treatment Strategies

Indication for treatment as a maximum diameter < 20 mm and absence of turbulent flow on duplex ultrasound were explicitly reported only in one study [3]. However, the mean aneurysm diameter in the surgical group was 26.3 mm, compared to a mean of 20.75 mm in the non-operated group. Surgical treatment was performed in 119 patients (68.8%), with two more patients (1.2%) additionally receiving cava filters. The remaining 54 (31.2%) were initially managed conservatively, but 5 patients (9.3%) required surgical intervention during follow-up.

**Figure 3:**
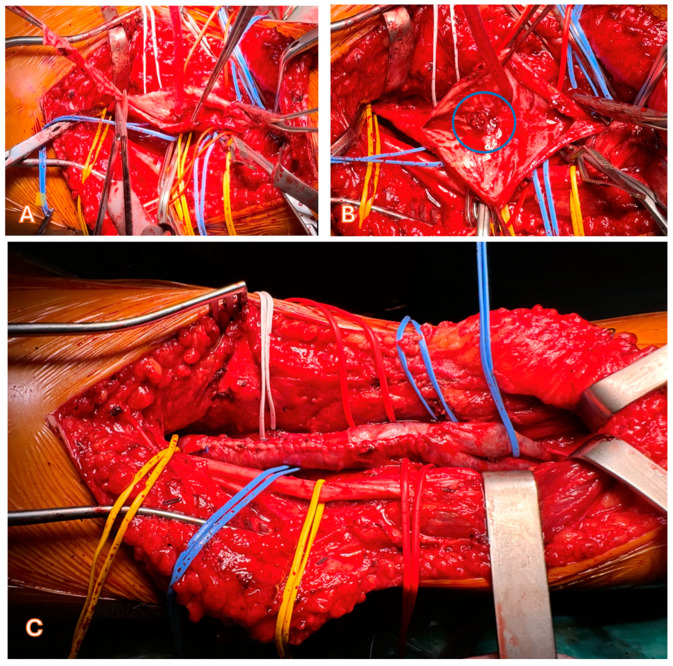
(**A**). Intra-operative view of the intraluminal thrombus (circle) and numerous collaterals. (**B**). Intra-operative view showing the resection of the exceeding venous wall. (**C**). Intra-operative view showing the results of surgery.

Surgical techniques included aneurysmectomy with lateral venorrhaphy in 73 patients (61.3%) (Figure 3), plication in 19 (16.0%), ligation in 6 (5.0%), resection with end-to-end anastomosis in 4 (3.4%), patchplasty using the great saphenous vein in 7 (5.9%), venous bypass in 9 (7.6%), and resection with vein transposition in 1 patient (0.8%).

No cases endovascularly treated were reported (Table 4).

The postoperative anticoagulation regimen varied across studies: some patients received unfractionated heparin for 2–3 days followed by warfarin for 3 months (20 patients) or low molecular weight heparin for 3 weeks to 1 month, combined or not with compression stockings (20–30 mmHg). In 9 patients (5.2%), anticoagulation was continued for 3–12 months using warfarin or rivaroxaban, and 3 (1.7%) were maintained on lifelong therapy. One study reported a mean duration of antithrombotic treatment of 7.4 months. Postoperative compression therapy was routinely prescribed in several series.

### 3.6. Outcomes

No cases of pulmonary embolism occurred after diagnosis, and clinical success with resolution of symptoms was reported in all cases regardless of treatment.

Postoperative complications were reported in 15 patients (13.0%), including wound infections (4, 3.5%), hematomas (7, 6.0%), and nerve injury (4, 3.5%).

During follow-up (mean follow-up 35 months, range 1–262 months), thrombosis of the surgical reconstruction was observed in 1 patient (0.8%), and aneurysm recurrence was noted in 12 patients (10.4%), all having undergone surgical treatment. Among the 54 initially non-operated patients, 1 (1.9%) developed deep vein thrombosis.

Death occurred in 3 cases (5.5%) in the non-surgical group: 2 (3.7%) cases due to malignancy and 1 (1.9%) from myocardial infarction (Table 5).

## 4. Discussion

Popliteal vein aneurysms (PVAs) are rare vascular anomalies, with a reported prevalence between 0.18% and 0.20% in patients undergoing venous ultrasound. However, their real incidence is likely to be underestimated due to the high rate of asymptomatic cases [4]. PVAs may present with local symptoms such as leg pain or swelling but are frequently diagnosed only when complications such as pulmonary embolism (PE) or deep vein thrombosis (DVT) occur [17]. Their etiology remains unclear. Some hypotheses have been proposed in the literature, including prior trauma, inflammation, and congenital weakness of the venous wall [13]. Histopathological findings reported by Noppeney described fibrosis of the aneurysmal wall, partial calcification, reduced intimal thickness, and increased collagen in the media [3]. Mendes observed a nonspecific inflammatory infiltrate in all specimens examined [13]. Only one study (Beaulieu et al.) reported a recurrence later diagnosed as Klippel-Trénaunay syndrome, whereas no consistent association with congenital venous malformations was identified in the remaining literature [18]. In our review, a history of previous trauma was reported in 8 patients (4.6%), representing the only potential etiologic factor clearly documented. No other associated vascular anomalies were found, suggesting that most PVAs may arise as isolated lesions. All patients were diagnosed using duplex ultrasound, which remains the first-line imaging modality. In some studies, preoperative phlebography was used to better define venous anatomy and plan surgical intervention. Despite some authors reporting higher rates (up to 40–63%) in individual series, our pooled data showed a lower overall prevalence (15.0%) of intraluminal thrombus across the included studies. The presence of thrombus was considered a risk factor for embolic events, although not all studies quantified this correlation [17]. In our analysis, pulmonary embolism occurred in 21 patients (12.1%), but due to inconsistent reporting, a direct association with thrombus presence could not be conclusively established. Currently, no standardized treatment guidelines exist for PVAs. Nonetheless, surgical intervention was the most frequent approach (68.8% of reported cases). Conservative management was adopted typically in case of smaller aneurysms and absence of turbulent flow [3]. Anticoagulation alone was used in some uncomplicated cases, although its effectiveness appeared limited, particularly in patients with embolic events. Based on our findings, surgical treatment should be considered in all aneurysms exceeding 20 mm in diameter and in patients with PE, thrombus, or progressive symptoms. The most common surgical technique was tangential aneurysmectomy with lateral venorrhaphy, despite other approaches that resulted in being efficacious as well. Indeed, surgical outcomes were generally favorable, with low complication rates and no reported postoperative pulmonary embolism or mortality. Postoperative anticoagulation emerged as a critical aspect of management. Although treatment protocols varied, most authors recommended continuing anticoagulation for 3 to 6 months after surgery to support endothelial healing and reduce the risk of graft or suture-line thrombosis. Some patients received prolonged or lifelong anticoagulation in cases of recurrent embolism or associated risk factors [4,18]. Twelve surgical patients (10.1%) experienced aneurysm recurrence during follow-up, but overall complication rates were low, including isolated cases of nerve injury, wound complications, and one graft thrombosis. No deaths were reported following diagnosis or treatment, supporting the safety of operative management in well-selected patients. Importantly, PVAs should be included in the differential diagnosis of pulmonary embolism, especially in patients without clear predisposing factors or when no embolic source is identified on standard imaging. Early recognition and targeted evaluation of the lower extremity venous system may be crucial for preventing potentially life-threatening events.

## 5. Conclusions

Popliteal venous aneurysms are rare vascular anomalies associated with a risk of pulmonary embolism. Based on our systematic review, surgical intervention combined with postoperative anticoagulation appears to provide favorable outcomes, with low rates of postoperative complications and recurrence. Conservative management may be considered in selected cases, particularly for small, asymptomatic aneurysms, but requires careful follow-up. Further prospective studies are needed to better define the natural history and optimal management strategies for this rare condition.

## 6. Limitations

This study has several limitations. The number of included studies and patients is relatively small, which limits the generalizability of the findings. Although one study was rated as good quality, the majority (7 out of 9) were of fair methodological quality, and one was rated as poor. Most studies lacked control groups and standardized designs, introducing potential biases in outcome interpretation. Significant heterogeneity was observed in terms of surgical techniques, inclusion criteria, and outcome reporting, which precluded a pooled quantitative analysis. Moreover, a risk of publication bias cannot be excluded, as negative or unsuccessful cases may be underreported. Despite these limitations, a key strength of this review lies in the systematic and rigorous methodological approach, including the use of NIH quality assessment tools appropriate to each study design and adherence to PRISMA guidelines through a pre-registered protocol.

## Figures and Tables

**Table 1:** PICO framework.

PICO ELEMENTS	Keywords	Search Terms
**P (Patient or Population)**	Patients with popliteal vein aneurysm	Popliteal venous aneurysms; lower limb venous aneurysms; venous aneurysms.
**I (Intervention)**	Aneurism resection and reconstruction	Aneurysmorraphy; Lateral venorraphy; plication; aneurysm resection; vein graft.
**C (Comparison)**	Conservative management, anticoagulant therapy	Anticoaulant therapy; compression therapy.
**O (Outcomes)**	Deep vein thrombosis, patency of the vascular recontruction, pulmonary embolism	Pulmonary embolism; Deep vein thrombosis; recurrence; survival.

**Table 2:** Quality Assessment.

Study	1	2	3	4	5	6	7	8	9	10	11	12	13	14	Total Yes	Quality
**Sessa** [1]	yes	yes	NR	Yes	NR	Yes	Yes	NA	Yes	No	Yes	NR	Yes	NR	8 yes	fair
**Noppeney** [3]	Yes	yes	CD	yes	No	yes	Yes	Yes	yes	Yes	yes	NR	NR	NR	9 yes	fair
**Fernandez** [10]	yes	yes	NR	yes	Yes	NR	yes	Yes	NA	Yes	NR	Yes	NR	NR	8 yes	fair
**Johnstone** [11]	yes	yes	NR	yes	NR	yes	yes	NA	yes	No	Yes	NR	NA	NR	7 yes	fair
**Zhao** [12]	yes	yes	NR	yes	Yes	Yes	NR	Yes	Yes	NA	NA	NA	NA	NA	7 yes	good
**Mendes** [13]	yes	yes	NR	yes	NR	yes	yes	NA	yes	NR	yes	NR	NR	NR	7 yes	fair
**Patel** [14]	yes	yes	CD	yes	NR	yes	yes	No	yes	No	yes	NA	CD	NR	7 yes	fair
**Beaulieu** [15]	yes	yes	NR	yes	NR	yes	NA	NA	yes	NA	yes	NR	NR	NR	6 yes	poor
**Donaldson** [16]	yes	yes	NR	yes	Yes	NR	yes	Yes	NA	Yes	NR	Yes	NR	NR	9 yes	fair

**Table 3:** Overview of the included studies.

N	Study	Year	N. Patients	Mean Age	M	W	Conservative Treatment	Surgical Treatment	Follow Up (Months)
**1**	**Sessa [1]**	2000	25	59	5	20	0	25	63
**2**	**Noppeney [3]**	2019	39	57.4	6	33	10	29	57.9
**3**	**Fernandez [10]**	2013	4	61	3	1	0	4	66
**8**	**Johnstone [11]**	2015	8	38.6	5	3	0	8	26
**9**	**Zhao [12]**	2017	7	55.8	5	2	4	3	24
**6**	**Mendes [13]**	2023	19	57.7	UN	UN	7	12	32
**4**	**Patel [14]**	2022	40	54	UN	UN	16	24	27
**5**	**Beaulieu [15]**	2020	10	52.9	5	5	0	10	32.2
**7**	**Donaldson [16]**	2014	21	58	12	9	17	4	38

M Men; W Women; UN unreported.

**Table 4:** Summary of baseline data in the included population.

	Total (n 173)	%
**Sex**		
Male	41	23.7
Female	73	42.2
Not reported	59	34.1
**Age**	56 (range 18–86 year)	
**Size of the PVA**		
Conservative treatment	20.7 mm	
Surgical treatment	26.3 mm	
**Comorbidities**		
Hypertension	30	17.3
Heart disease	6	3.5
Diabetes	3	1.7
Chronic vein insufficiency	78	45
Previous varicose vein surgery	7	4
Previous knee trauma	8	4.6
Deep venous thrombosis	11	6.4
**Clinical presentation**		
Asymptomatic	20	11.5
Pulmonary embolism	21	12.1
Edema or swelling	27	15.6
Lower limb pain	31	17.9
Discomfort of the limb	4	2.3
Popliteal mass	1	0.6
**Surgical treatment**		
Aneurysmectomy with lateral venorrhaphy	73	61.3
Plication	19	16
Ligation	6	5
Resection with end-to-end anastomosis	4	3.4
Patchplasty using the GSV	7	5.9
Venous bypass	9	7.6
Vein transposition	1	0.8

**Table 5:** Summary of outcomes and complications in the included population.

	Total (n 173)	%
30-day mortality	0	0
Long-term mortality	3	5.5
**Clinical Success**	173	100
**Post-operative complications**	15	13
Wound infections	4	3.5
Hematoma	7	6
Nerve injury	4	3.5
**Patency rate**	172	99.4
**Recurrence rate**	12	10.4
*Data are reported as n (%)*		

## Data Availability

This study is a systematic review based on previously published articles. No new data were generated, and all data supporting the findings are available within the cited publications.

## Supplementary Materials

The following supporting information can be downloaded at: https://www.mdpi.com/article/10.3390/jcm14103296/s1, Table S1: PRISMA checklist (Moher et al. 2009) [7].

## References

[1] Sessa C., Nicolini P., Perrin M., Farah I., Magne J.L., Guidicelli H. (2000). Management of symptomatic and asymptomatic popliteal venous aneurysms: A retrospective analysis of 25 patients and review of the literature. J. Vasc. Surg..

[2] McDevitt D.T., Lohr J.M., Martin K.D., Welling R.E., Sampson M.G. (1993). Bilateral Popliteal Vein Aneurysms. Ann. Vasc. Surg..

[3] Noppeney T., Kopp R., Pfister K., Schierling W., Noppeney J., Cucuruz B. (2019). Treatment of popliteal vein aneurysms. J. Vasc. Surg. Venous Lymphat. Disord..

[4] Bergqvist D., Björck M., Ljungman C. (2006). Popliteal Venous Aneurysm—A Systematic Review. World J. Surg..

[5] Richardson W.S., Wilson M.C., Nishikawa J., Hayward R.S. (1995). The well-built clinicalquestion: A key to evidence-based decisions. ACP J. Club.

[6] Page M.J., Moher D., Bossuyt P.M., Boutron I., Hoffmann T.C., Mulrow C.D., Shamseer L., Tetzlaff J.M., Akl E.A., Brennan S.E. (2021). PRISMA2020 explanation elaboration: Updated guidance exemplars for reporting systematic reviews. BMJ.

[7] Moher D., Liberati A., Tetzlaff J., Altman D.G., PRISMA Group (2009). Preferred reporting items for systematic reviews and meta-analyses: The PRISMA statement. BMJ.

[8] National Institutes of Health (2020). Quality Assessment of Controlled Interventionstudies. https://www.nhlbi.nih.gov/health-topics/study-quality-assessment-tools.

[9] Sommer A.E., Golden B.P., Peterson J., Knoten C.A., O’Hara L., O’Leary K.J. (2018). Hospitalized patients’ knowledge of care: A systematic review. J. Gen. Intern. Med..

[10] Maldonado-Fernandez N., Lopez-Espada C., Martinez-Gamez F.J., Galan-Zafra M., Sanchez-Maestre M.L., Herrero-Martinez E., Mata-Campos J.E. (2013). Popliteal venous aneurysms: Results of surgical treatment. Ann. Vasc. Surg..

[11] Johnstone J.K., Fleming M.D., Gloviczki P., Stone W., Kalra M., Oderich G.S., Duncan A.A., De Martino R.R., Bower T.C. (2015). Surgical treatment of popliteal venous aneurysms. Ann. Vasc. Surg..

[12] Zhao S., Wang X., Sheng H., Huang W., Zhu Y. (2017). Our experience of symptomatic and asymptomatic popliteal venous aneurysm. J. Vasc. Surg. Cases Innov. Tech..

[13] Azevedo Mendes D., Machado R., Veiga C., Almeida R. (2023). Institutional Experience with Venous Aneurysms—Insights on the Natural History and Outcomes of Surgical Treatment. Port. J. Card. Thorac. Vasc. Surg..

[14] Patel R., Woo K., Wakefield T.W., Beaulieu R.J., Khashram M., De Caridi G., Benedetto F., Shalhub S., El-Ghazali A., Silpe J.E. (2022). Contemporary management and outcomes of peripheral venous aneurysms: A multi-institutional study. J. Vasc. Surg. Venous Lymphat. Disord..

[15] Beaulieu R.J., Boniakowski A.M., Coleman D.M., Vemuri C., Obi A.T., Wakefield T.W. (2021). Closed plication is a safe and effective method for treating popliteal vein aneurysm. J. Vasc. Surg. Venous Lymphat. Disord..

[16] Donaldson C.W., Oklu R., Watkins M.T., Donaldson M.C., Abtahian F., Schainfeld R.M., Jaff M.R., Weinberg I. (2014). Popliteal venous aneurysms: Characteristics, management strategies, and clinical outcomes—A modern single-center series. Ann. Vasc. Surg..

[17] Nasr W., Babbitt R., Eslami M.H. (2008). Popliteal Vein Aneurysm: A Case Report and Review of Literature. Vasc. Endovasc. Surg..

[18] Aldridge S.C., Comerota A.J., Katz M.L., Wolk J.H., Goldman B.I., White J.V. (1993). Popliteal venous aneurysm: Report of two cases and review of the world literature. J. Vasc. Surg..

